# Highly Efficient Peroxymonosulfate Electroactivation on Co(OH)_2_ Nanoarray Electrode for Pefloxacin Degradation

**DOI:** 10.3390/nano14151312

**Published:** 2024-08-04

**Authors:** Tonghui Bao, Hui Ke, Wanjiang Li, Linke Cai, Yi Huang

**Affiliations:** Engineering Research Center of Photoenergy Utilization for Pollution Control and Carbon Reduction, Ministry of Education, College of Chemistry, Central China Normal University, Wuhan 430079, China; thbao123@mails.ccnu.edu.cn (T.B.); jczhang@mails.ccnu.edu.cn (H.K.); lwj5596@mails.ccnu.edu.cn (W.L.); cailinke@mails.ccnu.edu.cn (L.C.)

**Keywords:** peroxymonosulfate, electrochemistry, Co(OH)_2_, wastewater treatment, pefloxacin

## Abstract

The activation of PMS to produce active species is an attractive technique for antibiotic degradation but is restricted to the low reaction kinetics and high costs. In this work, a cobalt-based catalyst was prepared by in situ electrodeposition to enhance the electrically activated PMS process for the degradation of antibiotics. Almost 100% of pefloxacin (PFX) was removed within 10 min by employing Co(OH)_2_ as the catalyst in the electrically activated peroxymonosulfate (PMS) process, and the reaction kinetic constant reached 0.52 min^−1^. The redox processes of Co^2+^ and Co^3+^ in Co(OH)_2_ catalysts were considered to be the main pathways for PMS activation, in which ^1^O_2_ was the main active species. Furthermore, this strategy could also achieve excellent degradation efficiency for other organic pollutants. This study provides an effective and low-cost strategy with no secondary pollution for pollutant degradation.

## 1. Introduction

Treatment of medical wastewater containing antibiotics has received worldwide attention [1,2]. Pefloxacin (PFX), a fluoroquinolone, is widely used in treating viral and bacterial infections [3,4,5]. However, antibiotics have been discharged into aquatic ecosystems through wastewater, animal excrement, and soil erosion, which has seriously threatened both human and ecological health [6,7]. Due to the antibacterial activity, water solubility, and stability of PFX, traditional methods such as biological processes, flocculation, adsorption, and membrane processes are inadequate or ineffective for their removal [8,9]. Therefore, it is necessary to develop new processes for efficiently removing PFX and other fluoroquinolones antibiotics.

Advanced oxidation processes (AOPs) represented by activated peroxymonosulfate (PMS) have been widely studied due to its effective treatment, broad applicability, and low environmental impact [10,11]. PMS activation produces reactive species such as hydroxyl radicals (•OH), sulfate radicals (SO_4_^•−^), and singlet oxygen (^1^O_2_) that have exhibited high potential for the removal of persistent organic pollutants and pharmaceuticals [12,13]. The activation of PMS can be achieved by many methods, such as ultraviolet, electricity, ultrasound, heat, and external catalyst activation [14,15]. Compared to other methods, metal ion activation strategies have been widely used due to their low energy consumption, simple operation, and high efficiency [16]. Meanwhile, Co^2+^ ions are regarded as the most reactive and efficient homogeneous catalyst for the activation of PMS, and the mechanism of activation of PMS is as follows [17,18]:

Co^2+^ + HSO_5_^-^ → Co^3+^ + SO_4_^•-^ + OH^-^ (1)

Co^3+^ + HSO_5_^−^ + OH^−^ → Co^2+^ +SO_5_^•−^ + H_2_O (2)

However, the activation of PMS based on Co^2+^ ions has the disadvantages of a high ionic demand and strong pH dependence [19]. Recently, non-homogeneous catalysts represented by transition metal oxides have attracted extensive attention due to their high catalytic activity and stable crystalline structure [11,20,21]. Unfortunately, the catalyst particles dispersed in the solution are also prone to metal ion leaching, which cause secondary contamination and biotoxicity [22].

Electrochemistry is considered an effective way to enhance catalyst performance and has been widely used in the Fenton catalytic process [23]. The electric fields can accelerate electron transfer between electron donors and acceptors, which enhances PMS activation (Equation (3)) [24].

HSO_5_^−^ + e^−^ → SO_4_^•−^ + OH^−^ (3)

On this basis, the combination of electrical activation and catalyst activation is a promising method for PMS activation, which not only avoids the release of toxic metal ions but also reduces the cost of catalyst [25,26]. Meanwhile, Jing et al. prepared NiCo_2_O_4_ nanoarray electrodes on a cathode for electroactivated PMS to remove rhodamine B (100 ppm), achieving 99.7% removal efficiency within 60 min [27]. Zhang et al. prepared a MnFe_2_O_4_ electrode for electroactivated PMS to promote the degradation of bisphenol A (10 ppm), achieving 100% removal efficiency within 90 min [28]. Therefore, the combination of heterogeneous and electrochemistry activation of PMS is expected to present economical and efficient degradation of antibiotics.

Considering the excellent PMS activation performance of cobalt-based catalysts, we prepared a Co(OH)_2_ catalyst applied to the electrocatalytic system to realize the efficient removal of antibiotics by the activation of PMS. In this study, electrodes from a Co(OH)_2_ nanosheet array grown on Ti mesh (Co(OH)_2_@Ti) were prepared for electrochemically activated PMS to remove PFX, and we investigated the effects of pH, PMS concentration, and voltage on the degradation of PFX in this system. Then, the types and contributions of the active species produced by PMS electroactivation were identified through electron spin resonance (ESR) and free radical quenching experiments. Finally, the mechanism of PFX degradation via the Co(OH)_2_@Ti/electroactivated PMS (Co(OH)_2_@Ti/EA-PMS) process was proposed.

## 2. Materials and Methods

### 2.1. Preparation of Co(OH)_2_@Ti Nanoarray Electrode

The materials and chemicals utilized are shown in the Supporting Information of Table S1. The Co(OH)_2_@Ti nanoarray electrodes were prepared by electrochemical in situ deposition. Ti mesh (2 cm × 2 cm) was used as the working electrode, a Pt sheet was used as the counter electrode, and 0.025 mol/L cobalt nitrate (Co(NO_3_)_2_) was the electrode solution, which were reacted at −1.0 V (vs. Ag/AgCl) for 240 s. After the reaction, the Ti mesh was repeatedly cleaned and dried to obtain the Co(OH)_2_@Ti nanoarray electrodes.

### 2.2. Experimental Setups

The Co(OH)_2_@Ti/EA-PMS process was carried out using a standard three-electrode electrochemical cell, with the Co(OH)_2_@Ti as the working electrode, Ag/AgCl as the reference electrode, and Pt mesh as the counter electrode. In the experiment of removing PFX by activating PMS, 30 mL of the mixed solutions (0.1 mol/L K_2_SO_4_, 10 ppm PFX) was first added to the electrolytic cell. Then, a specific amount of PMS was introduced, and an electrochemical reaction was initiated by applying a constant voltage. Meanwhile, argon gas was continuously introduced into the solution during the reaction. Each electrochemical test was performed in triplicate.

### 2.3. Analytical Methods

The catalyst was characterized and analyzed through scanning electron microscopy (SEM, JSM-7610E, JEOL, Tokyo, Japan), transmission electron microscopy (TEM, JEM 2100F, JEOL, Tokyo, Japan), energy-dispersive X-ray spectroscopy (EDS, Oxford, UK), X-ray diffraction (XRD, Rigaku Ultima IV, Bruker, Saarbrucken, Germany), and X-ray photoelectron spectroscopy (XPS, K-Alpha, Thermo Scientific Waltham, MA, USA). The linear sweep voltammetry (LSV) test was carried out with a CHI 760E (Shanghai Chenhua Instrument Company, Shanghai, China). The PFX, RhB, and TC concentrations were determined by using an ultraviolet–visible spectrophotometer (UV-5500PC, Shimadzu, Kyoto, Japan), and the standard curves are shown in Figure S1. The electron spin resonance (ESR, A300, Bruker, Saarbrucken, Germany) was employed with DMPO and TEMP as spin-trapping agents for the detection of reactive species during the activation of PMS.

The PFX degradation efficiency and the pseudo-first-order rate constant were calculated using Equations (1) and (2).

(4) $$\mathrm{Degradation}\;\mathrm{efficiency}\;\left(\%\right)=\frac{C_{0}-C_{t}}{C_{0}}\times 100\%$$

(5) $$\;\;\;\;\;\;\;\;\;\;\frac{C_{t}}{C_{0}}=exp\left(-kt\right)$$

where C_0_ and C_t_ are the concentrations of PFX at 0 min and t min, respectively; and k (min^−1^) is the pseudo-first-order rate constant.

## 3. Discussion

### 3.1. Characterization of Catalytic Electrodes

**Figure 1:**
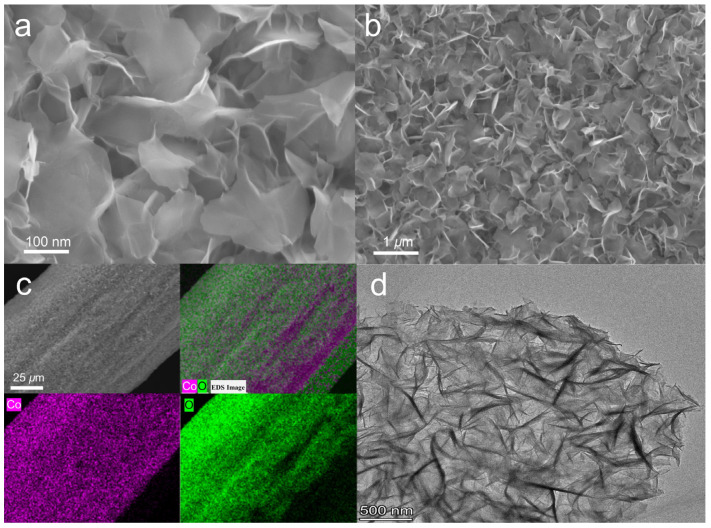
Morphological characterization of Co(OH)_2_@Ti nanoarray electrodes: (**a**,**b**) SEM images at different magnifications. (**c**) EDS images and (**d**) TEM images.

The morphology of the Co(OH)_2_@Ti electrode surface was first evaluated through SEM analysis. As shown in Figure 1a, b, the Ti mesh surface formed a uniformly distributed petal-like structure, which provided more active sites. Correspondingly, the elemental mapping images of Co(OH)_2_@Ti in Figure 1c confirm the uniform distribution of Co and O elements on the Ti mesh. Subsequently, the high-resolution TEM image of the Co(OH)_2_ catalysts was obtained (Figure 1d), revealing the nanoflake micromorphology of the catalysts, which was consistent with the SEM images.

XRD analysis was performed to examine the composition and structure of the Co(OH)_2_. Meanwhile, the Co(OH)_2_ catalysts were collected on carbon paper to avoid interference from the Ti substrate. As shown in Figure S2a, the corresponding diffraction peak corresponding to the plane of the C substrate can be found at 26.5° and 54.5°, and distinct diffraction peaks at 19.0°, 32.4°, 37.8°, 51.3°, and 57.7° are observed, which closely correspond to Co(OH)_2_ (PDF#03-0913) [29]. The Raman spectrum displays that the peaks related to Co(OH)_2_ appear at 665, 506, and 463 cm^−1^ (Figure S2b). Notably, no impurity peaks appear in the XRD and Raman spectra, providing evidence of the successful deposition of a pure Co(OH)_2_ catalytic layer.

### 3.2. Degradation of PFX in Co(OH)_2_@Ti/EA-PMS Process

**Figure 2:**
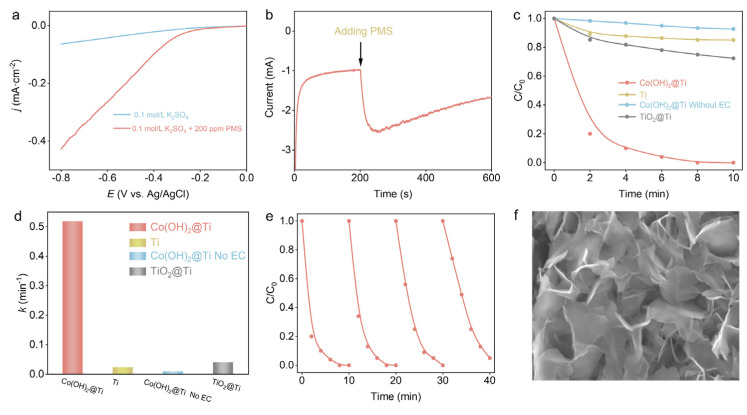
PFX removal performance and catalyst stability in Co(OH)_2_@Ti/EA-PMS process. (**a**) LSV curve in Co(OH)_2_@Ti/EA-PMS process. (**b**) Time–current curve at −0.8 V (V vs. Ag/AgCl) in Co(OH)_2_@Ti/EA-PMS process. (**c**) Removal efficiency and (**d**) first-order kinetic fitting constants of PFX for 10 min under different reaction conditions and systems. (**e**) Reusability of Co(OH)_2_ in Co(OH)_2_@Ti/EA-PMS system. (**f**) SEM images of Co(OH)_2_@Ti electrode after four-cycle experiments.

Firstly, the PMS activation performance of the Co(OH)_2_@Ti electrode was tested in 0.1 M K_2_SO_4_ with and without 200 ppm PMS electrolyte. In comparison to the scenario without PMS, the LSV curves show that the Co(OH)_2_ catalysts demonstrate a remarkable increase in current density in the presence of PMS (Figure 2a). Furthermore, the time–current curve of PMS activated by Co(OH)_2_ at the potential of −0.8 V (V vs. Ag/AgCl) was obtained, and the electrode solution was initially 0.1 M K_2_SO_4_, and 200 ppm PMS was added at 200 s (Figure 2b). Clearly, the current density increased rapidly with the addition of PMS. The above results jointly suggest the efficient electron transfer between Co(OH)_2_ and PMS, indicating the exciting potential of Co(OH)_2_ catalysts for PMS electroactivation.

To assess the catalytic performance of the Co(OH)_2_@Ti electrode, PFX was selected as a target pollutant. The degradation rate of PFX over time is presented in Figure 2c, with an initial concentration of 10 mg L^–1^. The Co(OH)_2_@Ti/EA-PMS process achieved complete removal of PFX within 10 min. In contrast, only 27.6% and 35.0% of PFX was degraded via the Ti/EA-PMS process and the TiO_2_@Ti/EA-PMS process, respectively. Additionally, cobalt-based catalysts activate PMS poorly in the absence of electroactivation. Employing pseudo-first-order reaction kinetics (Figure 2d), the reaction rate constant on the Co(OH)_2_@Ti electrode was determined to be 0.52 min^–1^, which was significantly higher than that on the Ti electrode (0.04 min^−1^) and the TiO_2_@Ti electrode (0.02 min^–1^). Meanwhile, the Co(OH)_2_@Ti electrode exhibited excellent PMS activation potential, achieving 90% activation of the PMS within 10 min (Figure S3). These results verify that the Co(OH)_2_@Ti electrode has an inspiring potential for the removal of PFX by activating PMS. Compared to other catalytic systems, the Co(OH)_2_@Ti/EA-PMS process not only efficiently removes organic pollutants but also significantly reduces catalyst costs (Table S2). Furthermore, the reusability of Co(OH)_2_ in the Co(OH)_2_@Ti/EA-PMS system was evaluated by four-cycle experiments. The PFX degradation efficiency under four consecutive reactions was 100%, 100%, 100%, and 95% (Figure 2e). The slight decrease in the catalytic activity of Co(OH)_2_ may be attributed to the adsorption of intermediate products of PMS activation and PFX degradation on the surface of the catalysts to cover the active sites [30,31]. After four consecutive cycles, the Co(OH)_2_ catalysts maintained their nanoflake morphology, with almost no leaching of cobalt ions (determined by inductively coupled plasma mass spectrometry (ICP-MS)) from the electrode, indicating that the catalysts have inspiring cycling stability (Figure 2f).

**Figure 3:**
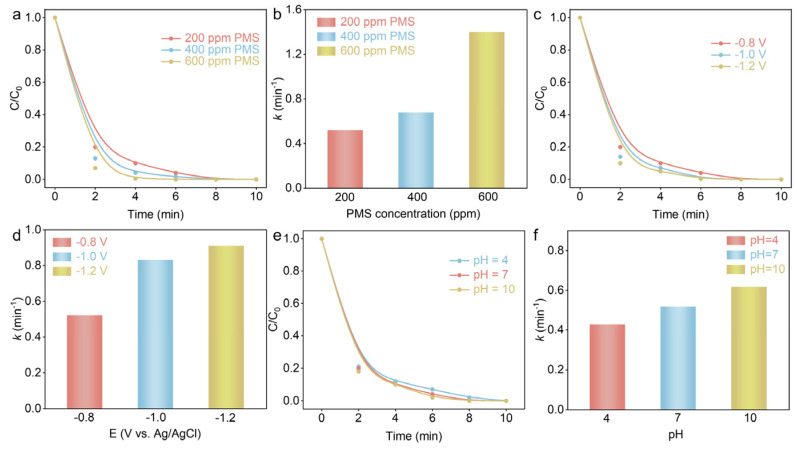
The degradation efficiency and kinetics of PFX in the Co(OH)_2_@Ti/EA-PMS process under different operating parameters. The effect of PMS concentrations on PFX degradation: (**a**) efficiency and (**b**) kinetics. The effect of voltage on PFX degradation: (**c**) efficiency and (**d**) kinetics. The effect of pH on PFX degradation: (**e**) efficiency and (**f**) kinetics.

**Figure 4:**
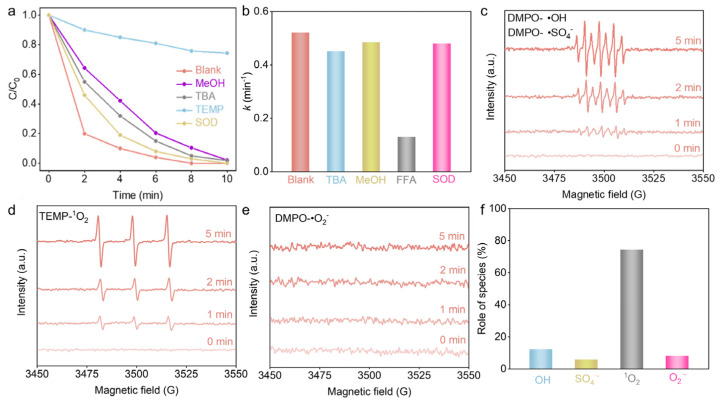
Mechanism of PFX degradation in Co(OH)_2_@Ti/EA-PMS system. (**a**) PFX degradation curves and (**b**) kinetics with addition of different quenching agents. EPR spectra of (**c**) DMPO-•OH and DMPO-SO_4_^•−^, (**d**) TEMP-^1^O_2,_ and (**e**) DMPO-•O_2_^−^ in Co(OH)_2_@Ti/EA-PMS process. (**f**) Proportion of four oxygen species in Co(OH)_2_@Ti/EA-PMS system.

In order to obtain the optimal experimental conditions (e.g., pH value, PMS concentration, and voltage) of Co(OH)_2_@Ti electroactivated PMS for the degradation of PFX, the impact of different PMS concentrations on the degradation of PFX in the Co(OH)_2_@Ti/EA-PMS system was first investigated (Figure 3a). As the concentration of PMS increased, the degradation rate of PFX increased due to the fact that the higher concentration of PMS produced more activated radicals (Figure 3b). Figure 3c shows the effect of different potentials (−0.8 V, −1.0 V, and −1.2 V (V vs. Ag/AgCl)) on the degradation of PFX. The degradation rate of PFX increased with the increase in voltage from −0.8 V to 1.2 V due to the applied voltage increase accelerating the activation of PMS, thus accelerating the PFX degradation efficiency (Figure 3d). Furthermore, the effects of different initial pH values (4, 7, and 10) on the degradation efficiency of PFX in the Co(OH)_2_@Ti/EA-PMS system were studied (Figure 3e and Figure 4f). When the initial pH value was 10, the degradation rate of PFX was the best, and the PFX degradation rate constant was 0.62 min^−1^, indicating that the weak alkaline environment was conducive to the degradation of PFX. It is noteworthy that PFX was also effectively degraded under weak acid and neutral conditions, confirming the wide pH range of the system.

### 3.3. The Mechanism of PFX Degradation in Co(OH)_2_@Ti/EA-PMS Process

Free radicals generated by PMS electroactivation play a crucial role in the degradation of PFX. Quenching experiments with scavengers were further conducted to clarify the contribution of different free radicals to the degradation of PFX. Meanwhile, furfuryl alcohol (FFA), tert-butanol (TBA), and superoxide dismutase (SOD) were used as the quenching agents for ^1^O_2_, •OH, and •O^2−^, respectively [32,33], and MeOH was used as the quenching agent for •OH and SO_4_^•−^ [34]. Employing pseudo-first-order reaction kinetics, the addition of TBA, MeOH, FFA, and SOD resulted in a reduction in the degradation rate constant of PFX by 13.5%, 6.9%, 75.2%, and 7.7%, respectively (Figure 4a,b). Therefore, we concluded that ^1^O_2_ was involved in PFX degradation as the main ROS produced by PMS activation. Additionally, we found that the degradation rate of PFX was higher in D_2_O compared to H_2_O due to the longer lifetime of ^1^O_2_ in D_2_O, which confirmed that that ^1^O_2_ is the main reactive oxygen species involved in PFX degradation (Figure S4) [35]. The DMPO and TEMP spin-trapping ESR was further used to determine the radical species involved in the Co(OH)_2_@Ti/EA-PMS process [36]. As shown in Figure 4c,d, the signals of SO_4_^•−^, •OH, and ^1^O_2_ were detected; however, the signal of •O^2−^ was not observed due to the extremely small reaction rate of DMPO with •O^2−^ and the short lifetime of DMPO-•O^2-^ (Figure 4e). In addition, the quantitative analysis of ^1^O_2_, SO_4_^•−^, •OH, and •O^2−^ radicals produced in the Co(OH)_2_@Ti/EA-PMS system was performed by the free radical molecular probe method using DPBF (1,3-Diphenylisobenzofuran), p-HBA (4-Hydroxybenzoic acid), TA-PL (terephthalic acid photoluminescence), and NBT (Nitro blue tetrazolium chloride), respectively [37,38,39,40]. As shown in Figure 4f, the contribution of the ^1^O_2_, SO_4_^•−^, •OH, and •O_2_^−^ radicals to PFX degradation was 74.26%, 5.6%, 12.14%, and 8%, respectively, which was consistent with the free radical quenching experiments. The source of ROS was further determined by excluding PMS and O_2_ interference separately. As shown in Figure S5, the ROS were mainly derived from PMS activation, and different atmospheres had little effect.

**Figure 5:**
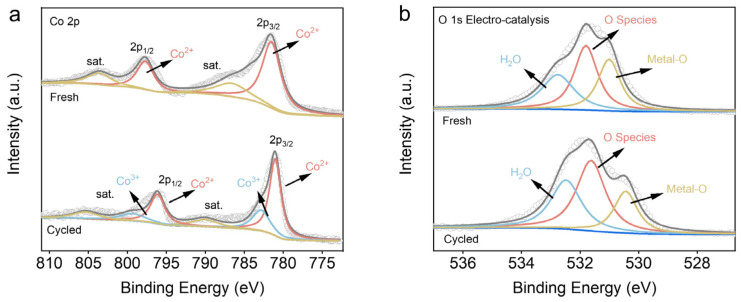
XPS spectrum of Co(OH)_2_@Ti nanoarray electrodes: (**a**) Co 2p and (**b**) O 1s before (up) and after (down) Co(OH)_2_@Ti/EA-PMS process.

To clarify the electroactivation mechanism of PMS by Co(OH)_2_@Ti, XPS was employed to analyze the catalyst chemical valence before and after the reaction. As shown in Figure 5a, the peaks at the binding energies of 781.6 eV and 797.8 eV are assigned to Co^2+^ 2p3/2 and Co^2+^ 2p1/2, respectively [41]. The peaks of Co 2p in Co(OH)_2_ exhibit a negative shift of 0.5 eV after the activation process of PMS compared to the initial Co(OH)_2_. The Co 2p spectra exhibit new peaks corresponding to Co^3+^, which indicates that the electron transfer resulting from the conversion from Co^2+^ to Co^3+^ was essential for the activation of PMS [42,43]. As shown in Figure 5b, the three characteristic peaks at 531.0 eV, 531.8 eV, and 532.8 eV in the O 1s spectrum are assigned to lattice oxygen, reactive oxygen, and H_2_O, respectively [44,45]. The ratio of reactive oxygen/lattice oxygen increases significantly from 1.33 to 2.2 after the PMS activation process, indicating the formation of substantial ROS during the activation of PMS [46,47].

Based on the above results and previous works, the PFX degradation mechanism in the Co(OH)_2_@Ti/EA-PMS system was proposed as follows [11,18,48,49]: Firstly, the hydroxyl group on the surface of Co^2+^ is displaced by the SO_5_^•−^ group, forming complexes. Subsequently, an internal nucleophilic attack leads to the formation of the intermediate Co^2+^-OO-Co^2+^ with Co^2+^ on adjacent surfaces, and O-O bond cleavage leads to Co^3+^, which is similarly stripped of the hydroxyl group on the surface to be replaced by the SO_5_^•−^ group, forming the intermediate Co^3+^-OO-Co^3+^. Finally, O-O bond cleavage leads to the formation of ^1^O_2_ and the regeneration of the active sites. The relevant reactions are represented by Equations (5)–(10):

Co^2+^-OH + HSO_5_^−^ → Co^2+^-SO_5_^−^ + H_2_O (6)

Co^2+^-SO_5_^−^ + Co^2+^-OH → Co^2+^-OO-Co^2+^ + H^+^ + SO_4_^2−^ (7)

Co^2+^-OO-Co^2+^ + 2H_2_O → 2Co^3+^-OH + 2HO^−^ (8)

Co^3+^-OH + HSO_5_^−^ → Co^3+^-SO_5_^−^ + H_2_O (9)

Co^3+^-OH + Co^3+^-SO_5_^−^ → Co^3+^-OO-Co^3+^ + H^+^ + SO_4_^2−^ (10)

Co^3+^-OO-Co^3+^ + 2H_2_O → 2Co^2+^-OH + 2H^+^ + ^1^O_2_ (11)

**Figure 6:**
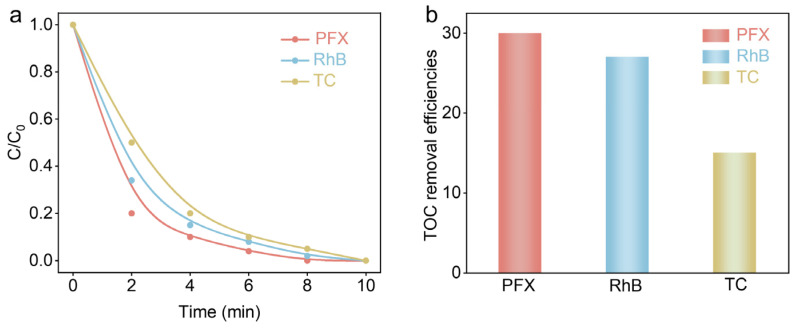
Other organic pollutants’ (**a**) degradation curves and (**b**) mineralization rates via Co(OH)_2_@Ti/EA-PMS process.

Consequently, we propose that the Co(OH)_2_@Ti/EA-PMS system could be extended to the treatment of other organic pollutants in wastewater. Using rhodamine B (RhB) and tetracycline (TC) as examples, we found that the degradation efficiency of RhB and TC was almost 100% in the Co(OH)_2_@Ti/EA-PMS process within 10 min (Figure 6a), and the mineralization rates were 27% and 14% for RhB and TC, respectively (Figure 6b). This result displays the potential practical applications of the Co(OH)_2_@Ti/EA-PMS process for treating various organic pollutants in wastewater.

## 4. Conclusions

In this study, Co(OH)_2_@Ti nanoarray electrodes were successfully fabricated by electrochemical in situ deposition. The catalysts were further characterized by XRD, Raman spectroscopy, SEM, TEM, and XPS to verify the petal-like structure of the Co(OH)_2_ uniformly loaded on the Ti mesh. In addition, the Co(OH)_2_@Ti electrode showed an inspiring potential for the removal of PFX by activating PMS, achieving 100% removal of PFX within 10 min at −0.8 V (V vs. Ag/AgCl). Combined with EPR and quenching experiments, we inferred that the PMS activation was mainly due to the electron transfer by redox processes of Co^2+^ and Co^3+^ in the Co(OH)_2_ catalysts, in which ^1^O_2_ was the main active species. On this basis, the PFX degradation mechanism in the Co(OH)_2_@Ti/EA-PMS system was further proposed. In a word, this work provides an efficient strategy for PFX degradation, and the prepared Co(OH)_2_@Ti nanoarray electrodes show remarkable stability and performance in activating PMS for the degradation of PFX.

## Data Availability

The data are contained within the article and Supplementary Materials.

## Supplementary Materials

The following supporting information can be downloaded at https://www.mdpi.com/article/10.3390/nano14151312/s1, Table S1: Chemicals and materials; Table S2: Comparison of catalytic performance with other systems; Figure S1: The standard curves of PFX, RhB, and TC. Figure S2: Structural characterization and electrochemical performance of Co(OH)_2_ catalysts. (a) XPS spectrum and (b) Raman image of Co(OH)_2_ catalysts. Figure S3: Activating PMS on Co(OH)_2_ nanoarray electrode. (a) Standard curve of PMS. (b) Degradation curve of PMS. Figure S4: Degradation of PFX under H_2_O and D_2_O solutions: (a) degradation curves; (b) degradation kinetics. Figure S5: Degradation of PFX under different conditions: (a) degradation curves; (b) degradation kinetics. (References [50,51,52,53] are cited in Supplementary Materials).
