# Supplementary material for: Highly Efficient Peroxymonosulfate Electroactivation on Co(OH)2 Nanoarray Electrode for Pefloxacin Degradation
Source: Nanomaterials (Basel). 2024 Aug 4;14(15):1312. doi: 10.3390/nano14151312 (PMC11314119; doi:10.3390/nano14151312)
Supplement: Supplementary file 1 [file nanomaterials-14-01312-s001.zip › nanomaterials-3135656-supplementary.pdf]

Supporting information

Highly Efficient Peroxymonosulfate Electroactivation on $Co(OH)_2$ Nanoarray Electrode for Pefloxacin Degradation

Tonghui Bao, Hui Ke, Wanjiang Li, Linke Cai and Yi Huang *

Engineering Research Center of Photoenergy Utilization for Pollution Control and Carbon Reduction, Ministry of Education, College of Chemistry, Central China Normal University, Wuhan 430079, China

* Correspondence: yihuang@ccnu.edu.cn

Table S1. Chemicals and materials.

Name	**Purity**	**Manufacturing company**
Potassium sulfate	AR	Sinopharm Chemical Reagent Co.
Ethanol	AR	
Pefloxacin	AR	
Peroxymonosulfate	AR	
Methyl alcohol	AR	
Superoxide dismutase	AR	
Tertiary butyl alcohol	AR	
5,5-Dimethyl-1-pyrroline-N-oxide	AR	
Cobalt(II) nitrate Hexahydrate	AR	
2,2,6,6-Tetramethylpiperidine	AR	
Hydrochloric acid	AR	
Acetone	AR	
Ti mesh		Alibaba Group Holding Limited
Carbon paper		

Table S2. Comparison of catalytic performance with other systems.

Catalysts	Catalysts dosage	Organic pollutants	Oxidant concentration	Solution pH	Reaction temperature	Removal efficiency	References
CuFe-S	30 mg/L	Tetracycline 50 mg/L	H_2O_2 100 mM	2.0	30 °C	30 min: 91.9%	[1]
Electroplating sludge	30 mg/L	Tetracycline 50 mg/L	H_2O_2 0.3 mM	3.0	30 °C	60 min: 98.8%	[2]
Cr-doped ferrite	0.4 g/L	Total cholesterol 110 mg/L	PS 5 mM	3.0	30 °C	120 min: 96%	[3]
NF/MXene-Co_3O_4	40 mg	1,4-dioxane 0.5 mM	PMS 4.0 mM	2.7	25 °C	20 h: 95%	[4]
CuO-Fe_2O_3/MXene	0.1 g/L	Atrazine: 10 mg/L	PMS 0.3 mM	6.4	-	60 min: 100%	[5]
Co@Ti_{2-x}	5.0 mg	Sulfamethoxazole: 5 μM	PMS 2.0 mM	6.95	25 °C	40 min: 99.1%	[6]
Cr_2O_3/BC	0.4 g/L	Tetracycline 40 mg/L	PS 10 mM	7.0	-	30 min: 90%	[7]
$Co(OH)_2$	0.12 mg/cm^2	PFX 10 mg/L	PMS 200 ppm	7.0	25 °C	10 min: 100%	This work

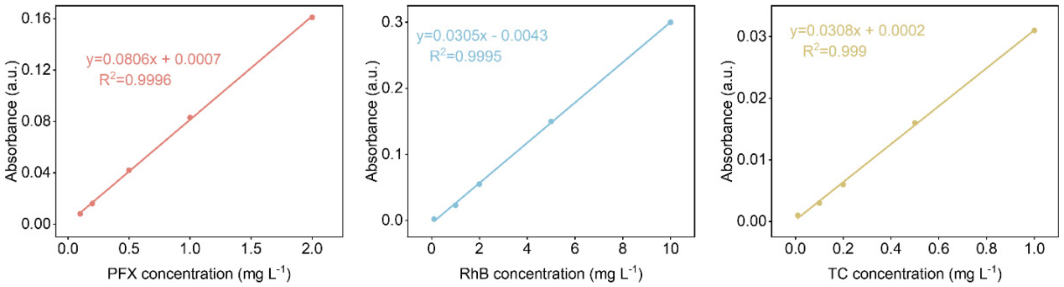

Figure S1. The standard curves of PFX, RhB and TC.

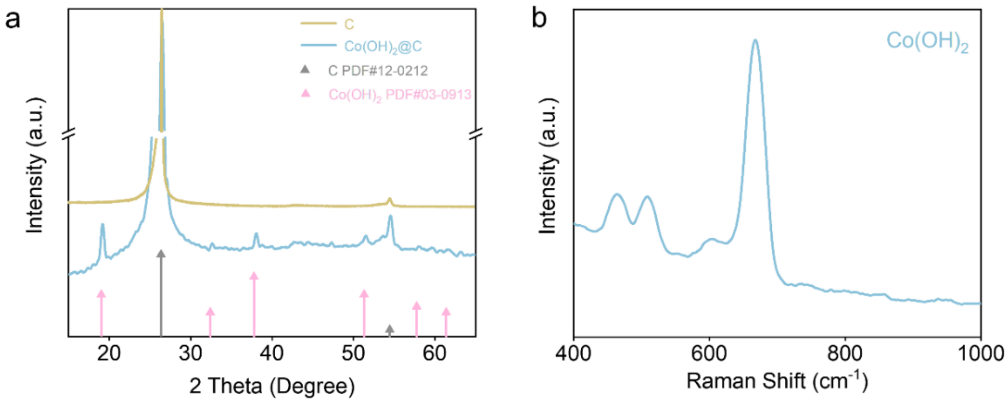

Figure S2. Structural characterization and electrochemical performance of the $Co(OH)_2$ catalysts. (a) XPS spectrum and (b) Raman image of $Co(OH)_2$ catalysts.

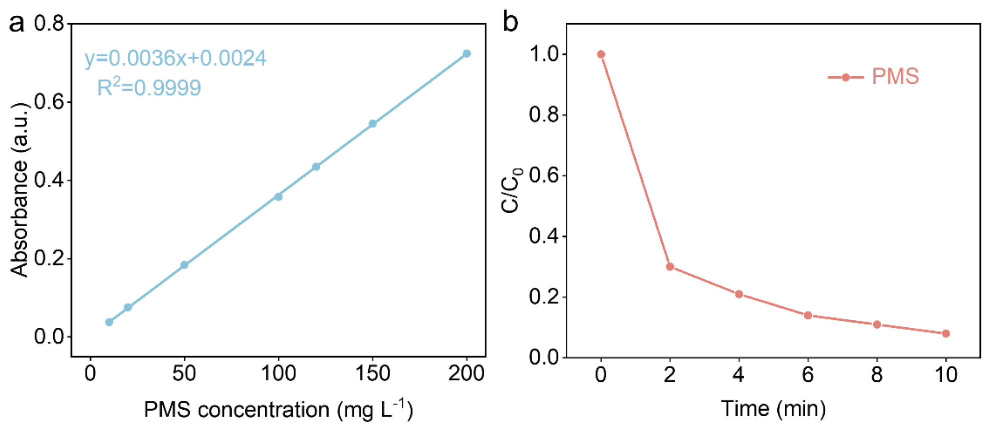

Figure S3. Activating PMS on $Co(OH)_2$ nanoarray electrode. (a) the standard curve of PMS (b) the degradation curve of PMS.

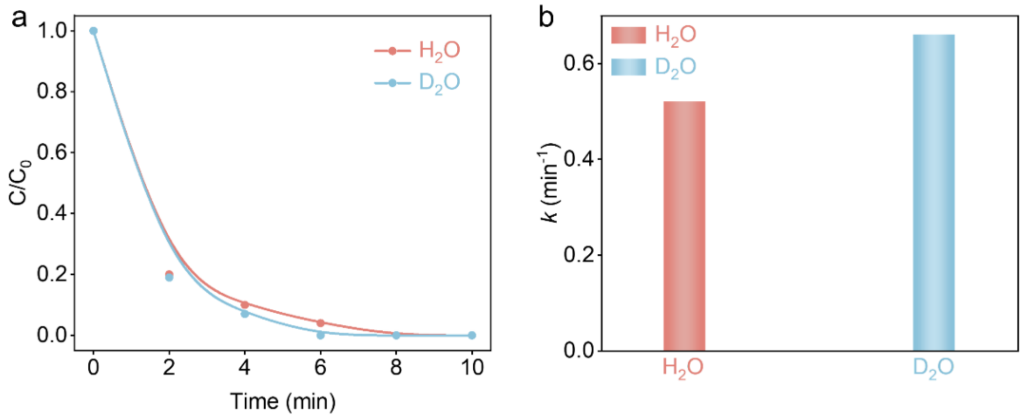

Figure S4. Degradation of PFX under H_2O and D_2O solutions: (a) degradation curves (b) degradation kinetics.

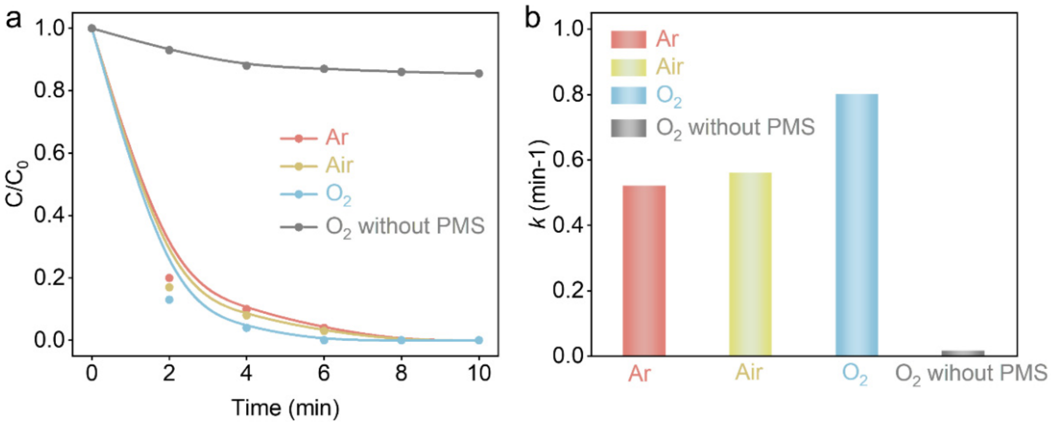

Figure S5. Degradation of PFX under different conditions: (a) degradation curves (b) degradation kinetics.

References

1. Zhou, Z.; Liu, T.; Wu, J.; Li, H.; Chu, S.; Zhu, X.; Zhang, L.; Lu, J.; Ivanets, A.; Davronbek, B.; et al. Preparation of copper-based catalysts from electroplating sludge by ultrasound treatment and their antibiotic degradation performance. *Environ. Res.* **2023**, *216*, 114567, doi:https://doi.org/10.1016/j.envres.2022.114567.
2. Zhou, Z.; Zhang, L.; Yan, B.; Wu, J.; Kong, D.; Romanovski, V.; Ivanets, A.; Li, H.; Chu, S.; Su, X. Removal of chromium from electroplating sludge by roasting-acid leaching and catalytic degradation of antibiotics by its residue. *J. Environ. Chem. Eng.* **2024**, *12*, 111754, doi:https://doi.org/10.1016/j.jece.2023.111754.
3. Xie, D.; Chu, S.; Zhang, S.; Ivanets, A.; Zhang, L.; Su, X. Facile synthesis of Cr-doped ferrite catalyst from Cr-containing electroplating sludge with activated persulfate for efficient degradation of tetracycline. *J. Environ. Chem. Eng.* **2022**, *10*, 108805, doi:https://doi.org/10.1016/j.jece.2022.108805.
4. Li, W.; Li, W.; He, K.; Tang, L.; Liu, Q.; Yang, K.; Chen, Y.-D.; Zhao, X.; Wang, K.; Lin, H.; et al. Peroxymonosulfate activation by oxygen vacancies-enriched MXene nano-Co3O4 co-catalyst for efficient degradation of refractory organic matter: Efficiency, mechanism, and stability. *J. Hazard. Mater.* **2022**, *432*, 128719, doi:https://doi.org/10.1016/j.jhazmat.2022.128719.
5. Xu, P.; Wang, P.; Li, X.; Wei, R.; Wang, X.; Yang, C.; Shen, T.; Zheng, T.; Zhang, G. Efficient peroxymonosulfate activation by CuO-Fe2O3/MXene composite for atrazine degradation: Performance, coexisting matter influence and mechanism. *Chem. Eng. J.* **2022**, *440*, 135863, doi:https://doi.org/10.1016/j.cej.2022.135863.
6. Song, H.; Du, R.; Wang, Y.; Zu, D.; Zhou, R.; Cai, Y.; Wang, F.; Li, Z.; Shen, Y.; Li, C. Anchoring single atom cobalt on two-dimensional MXene for activation of peroxymonosulfate. *Appl. Catal., B* **2021**, *286*, 119898, doi:https://doi.org/10.1016/j.apcatb.2021.119898.
7. Guo, L.; Zhao, J.; Zhao, L.; Tang, Y.; Zhou, J.; Shi, B. Persulfate activation by Cr2O3/BC derived from chrome shavings for antibiotics degradation. *Chem. Eng. J.* **2021**, *420*, 127698, doi:https://doi.org/10.1016/j.cej.2020.127698.
